# A new dataset for mongolian online handwritten recognition

**DOI:** 10.1038/s41598-022-27267-8

**Published:** 2023-01-02

**Authors:** Yuecai Pan, Daoerji Fan, Huijuan Wu, Da Teng

**Affiliations:** College of Electronic Information Engineering, Inner Mongolia University, Hohhot, 010021 China

**Keywords:** Engineering, Electrical and electronic engineering

## Abstract

This paper introduces a new traditional Mongolian word-level online handwriting dataset, MOLHW. The dataset consists of handwritten Mongolian words, including 164,631 samples written by 200 writers and covering 40,605 Mongolian common words. These words were selected from a large Mongolian corpus. The coordinate points of words were collected by volunteers, who wrote the corresponding words on the dedicated application for their mobile phones. Latin transliteration of Mongolian was used to annotate the coordinates of each word. At the same time, the writer’s identification number and mobile phone screen information were recorded in the dataset. Using this dataset, we propose an encoder–decoder Mongolian online handwriting recognition model with a deep bidirectional gated recurrent unit and attention mechanism as the baseline evaluation model. Under this model, the optimal performance of the word error rate (WER) on the test set was 24.281%. Furthermore, we present the experimental results of different Mongolian online handwriting recognition models. The experimental results show that compared with other models, the model based on Transformer could learn the corresponding character sequences from the coordinate data of the dataset more effectively, with a 16.969% WER on the test set. The dataset is now freely available to researchers worldwide. The dataset can be applied to handwritten text recognition as well as handwritten text generation, handwriting identification, and signature recognition.

## Introduction

Pattern recognition has contributed greatly to machine vision applications. Handwriting recognition falls under the umbrella of pattern recognition. Handwriting recognition is the technique by which a computer system can recognize characters and other symbols written by individuals using natural handwriting^1^. With the popularity of mobile phones and digital devices, more applications of handwriting recognition have emerged, such as the handwriting input method, signature recognition, and business card recognition. In the Inner Mongolia Autonomous Region, China, about 4 million people speak and write the traditional Mongolian language. However, owing to the lack of datasets, the development of Mongolian online handwriting recognition has been slow. Even though there are some reports on Mongolian online handwriting recognition, the relevant datasets have not been published, making the comparison and evaluation of different models or algorithms impractical. Undoubtedly, the performance of training and recognition highly depends on the quantity and quality of training samples through deep neural networks^2^. Thus, it is necessary to build a large online handwritten Mongolian word database for all researchers in this area.

The main features that distinguish Mongolian from other languages are as follows: as an agglutinative language, its vocabulary is vast, including millions of words, and letters are seamlessly connected from top to bottom. In Mongolian online handwriting recognition, to our knowledge, MRG-OHMW^3^ is the first publicly available database for online handwritten Mongolian. The main shortcoming of this dataset is that the vocabulary only covers 946 Mongolian words, which is too small for Mongolian, and the handwriting trajectories were collected by an Anoto pen on paper, making them different from trajectories written with fingers on a touch screen. Thus, we aim to build a large Mongolian word-level online mobile handwritten database to promote the development of related research and applications.

**Figure 1:**
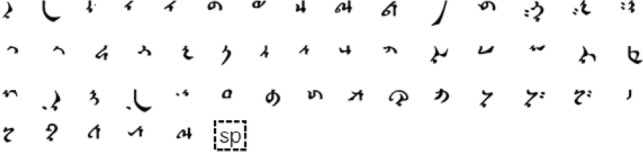
Grapheme code.

Therefore, this paper proposes a comprehensive Mongolian online handwritten dataset called “MOLHW”, which may be used as a benchmark dataset for the Mongolian online handwritten recognition task. The dataset was written by 200 people in total, and the data in it were collected by mobile phone application. The words were written using a finger on a touch screen. The vocabulary contained in MRG-OMHW^3^ consists of 946 words, while that in MOLHW is much larger, at 40,605 words. Considering that there are many ways to split and segment Mongolian words for analysis, in this paper, we choose the grapheme code as the Mongolian alphabet because it splits Mongolian in the most detailed way and carries no grammatical information. The grapheme code is shown in Fig.1 and contains 51 characters.

The MOLHW dataset is now freely available to researchers for various Mongolian online text-related applications, such as Mongolian online text recognition, handwritten text generation, writer identification and verification, and signature recognition. The main contributions of this paper can thus be summarized as follows.

- The creation of an open vocabulary benchmarking dataset of a Mongolian online handwritten dataset, MOHLW, which includes 164,631 samples written by 200 writers and covers 40,605 common Mongolian words.
- The development of tools, techniques, and procedures for Mongolian online text collection, verification, and transliteration.
- The development of a proposed benchmark model for recognition of online Mongolian handwritten words using the encoder–decoder model.
- A comparison of the performance of different models on this dataset.

The rest of the paper is organized as follows. “Related work” presents a literature review of Mongolian online text datasets. In “Overview of MOLHW”, we present the data collection steps used in this study and the dataset statistics. “Benchmark evaluation” details the data preprocessing and framing process. Additionally, this section presents three trained and validated models and the experimental results of Mongolian online character recognition algorithm using the MOHLW dataset. Then, for the experimental results of our baseline model, we provide the error analysis of the test set. Finally, we present the conclusions of this study in “Conclusion”.

## Related work

Currently reported Mongolian text recognition research can be divided into three categories: optical character recognition (OCR), historical document recognition, and handwriting recognition.

The segmentation method was adopted in the earliest Mongolian OCR. In the first step, the glyph is segmented from the image, then the feature of each glyph is extracted, and finally the glyph is classified and recognized by matching with the template^4^. In short, Mongolian characters are seamlessly connected, so the segmentation of glyphs is a difficult and challenging task. Therefore, the segmentation of characters will greatly affect the accuracy of recognition. Due to the above special nature of Mongolian, in recent years, many scholars have adopted the non-segmentation strategy^5–7^. Datasets for studying Mongolian OCR are relatively easy to obtain. Zhang et al.^5^ proposed a model based on sequence to sequence with an attention to recognize non-segmented printed Mongolian text in 2017, and the recognition accuracy of this experiment has reached 89.6%. The dataset used in this experiment belongs to the author and contains about 20,000 words and a total of 80,000 samples. In 2019, Wang et al.^6^ proposed end-to-end printed Mongolian text recognition based on bidirectional long short-term memory (BiLSTM) and connectionist temporal classification (CTC). The problem of Mongolian characters segmentation was not handled, but the author focused to the problem of sequence to sequence. The dataset consists of 800,000 samples collected from the dictionary and covers 20,250 words. In 2021, Cui et al.^7^ proposed a triplet attention Mogrifier network (TAMN) for irregular printed Mongolian text recognition. The TAMN network uses a special spatial transformation method to correct the distorted Mongolian image.The recognition accuracy reached 90.30% on their own dataset, which includes 98,085 Mongolian pictures from the China Mongolian News Network and covers 6538 words.

Research on the recognition of historical Mongolian documents has been conducted around Mongolian Kanjur—a Mongolian encyclopedia—the content of which involves religion, history, and literature. The main research point has been keyword spotting and the holistic recognition of the Woodblock–Print word^8–10^. The dataset used in Mongolian historical documents recognition is from scanned Mongolian Kanjur images, spanning about 200 pages.

The research on Mongolian handwriting recognition can be divided into two categories: offline and online handwriting recognition. The publication of online or offline handwriting datasets in various languages has contributed to the development of their respective handwriting techniques. For example, in the research of offline handwriting recognition, English has the CENPARMI^11^, CEDAR^12^, and IAM^13^ datasets; Chinese has the HCL2000^14^, CASIA Offline datasets^15^, and HIT-MW^16^ datasets; Japanese has the Kuchibue^17^ and Nakayosi^18^ datasets; and Arabic has IFN/ENIT^19^ and KHATT^20^. In Mongolian offline handwriting recognition, a word-level traditional Mongolian offline handwriting dataset (MHW) was developed by Daoerji^21^. The MHW dataset is divided into a training set and two test sets, including nearly 120,000 samples written by 200 different writers. The size of the training set, test set I, and test set II is 5000, 1000, and 939 words in the MHW dataset, respectively. The vocabularies of the training and testing set have a few intersections. Fan and Gao^22^ developed a hidden Markov model and deep neural network hybrid system to recognize offline handwritten Mongolian text, with an accuracy of 97.61% on MHW Test set I and an accuracy of 94.14% on MHW Test set II. In this work, the post-processing stage used the Viterbi algorithm on a dictionary that only contains a subset of the vocabulary of MHW that includes approximately 6734 words, and searched for the maximum possible result. Therefore, out-of-vocabulary (OOV) words could not be recognized. A lexicon-free conversational Mongolian offline handwritten recognition system with a two-dimensional recurrent neural network with CTC was proposed in^23^. The sub-word language model they used can recognize any word and showed the best performance, with word error rates (WERs) of 18.32% and 23.22% on MHW of the two test sets. In^24^, an encoder–decoder with an attention mechanism model for Mongolian offline handwritten recognition was proposed. The model consists of two LSTMs and one attention network. The first LSTM is an encoder that consumes a frame sequence of a one-word image. The second LSTM is a decoder that can generate a sequence of letters. The attention network is added between the encoder and decoder, which allows the decoder to focus on different positions in a sequence of frames during the decoding. The best accuracies on the two testing sets of MHW were 90.68% and 84.16%, respectively.

In the research of online handwriting recognition, a lot of online handwritten datasets have been published in various languages. For example, Arabic has the ADAB^25^, AltecOnDB^26^, and Online-KHATT^27^ datasets; Chinese has the CASIA Online datasets^15^; Mongolian has the MRG-OHMW^3^ datasets. In the development of Mongolian online handwriting recognition, Ma et al.^3^ published a database called “MRG-OHMW” for online handwritten Mongolian in 2016. There are 282,954 Mongolian word samples in the database, and their 300 writers come from a Mongolian ethnic minority. The vocabulary of this database covers 946 Mongolian words, with the word lenghth from one to fourteen Mongolian characters. The comparison between the MRG-OHMW database and our database is given in Table 1.

**Table 1:** Statistics of MRG-OHMW and MOLHW.

Database	Writers	Words	Maximum word length	Total size	Collection method
MRG-OHMW	300	946	14	282,954	Using Anoto pen on paper on paper
MOLHW	200	40,605	20	164,631	Writing with fingers on the touch screen

Ma et al. proposed a recognition model based on a CNN network and tested it on their own database with the 91.2% test accuracy. In 2016, Liu et al.^28^ proposed an online handwritten Mongolian word recognition method based on MWRCNN and position maps. On MRG-OHMW database, the method of combining multiple classifiers achieved the highest recognition accuracy of 93.24%. In 2017, Liu et al.^29^ proposed a five-bidirectional hidden-level deep bidirectional long short term memory (DBLSTM) network for online handwritten Mongolian word recognition. The best performance of the model adopted a noval sliding window method with the decoding method adopted the optimal path decoding is the word level recognition rate of 90.35% on the MRG-OHMW subset. In 2020, a new method, CMA-MOHR, for online handwritten Mongolian character recognition was proposed by Fan Yang et al.^30^. To evaluate the performance of the model, they carried out experiments of different models on their own dataset and got the best performance of 76.23% on their model. So far, however, their datasets have not been made public. Recently, the transformer network was proposed by Devlin^31^, which is completely based on the attention mechanism and recurrence and convolutions are not required in the whole network model. It is proved to be very effective in the field of handwriting recognition. In the field of online handwriting recognition, Matteo^32^ carried out experiments on an online handwritten dataset, released by STABILO, and the recognition model was based on transformer structure. The experimental results show that transformer is a significant breakthrough in the sequence to sequence problem.

## Overview of MOLHW

### Mongolian vocabulary selection

Like English, traditional Mongolian is a phonetic script, with 35 letters. Unlike letters in the Latin alphabet, Mongolian letters have different shapes depending on the position and context in a word. In Mongolian Unicode encoding, only 35 basic letters, called the Nominal Forms, are encoded, and there is no independent encoding for the different forms of each letter, called the Presentation Forms. Therefore, the text processing engine needs to display the correct glyphs according to the context. Because Mongolian Unicode encoding cannot represent unique glyphs, a Mongolian grapheme code set containing 51 elements was proposed by Fan et al.^22^. Compared to Unicode, grapheme codes have shown better performance on Mongolian handwritten recognition tasks. Therefore, in the MOLHW dataset, we provide both Unicode and grapheme codes labels at the same time.

**Figure 2:**
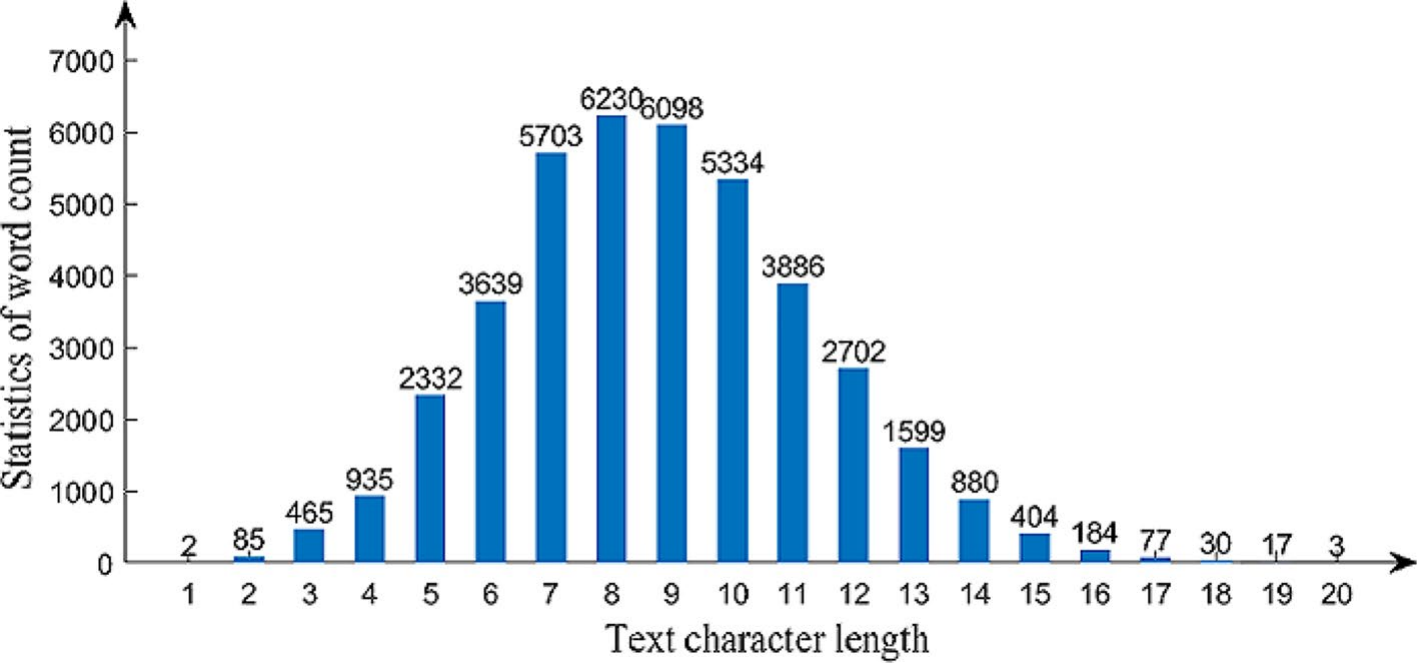
Word frequency statistics of different lengths.

Mongolian is considered to be one of the most morphologically complex languages. Mongolian words are formed by attaching suffixes to stems. One stem, especially a verb stem, can be used to generate dozens or hundreds of words by connecting different suffixes to it. According to incomplete statistics, the Mongolian vocabulary can reach one million words. Thus, it is impossible to cover all Mongolian words when building Mongolian-language datasets. To include all grammatical phenomena in Mongolian, we counted the word frequency from a Mongolian corpus containing more than 3 million words and selected 40,605 words as the vocabulary set of this dataset. The vocabulary set contains words with Unicode lengths from 1 to 20. We have counted the number of words of different lengths, and Fig. 2 shows the specific results It can be seen from the figure that the length of frequently used Mongolian words was concentrated in the range of 6–12 characters.

### Data collection

**Figure 3:**
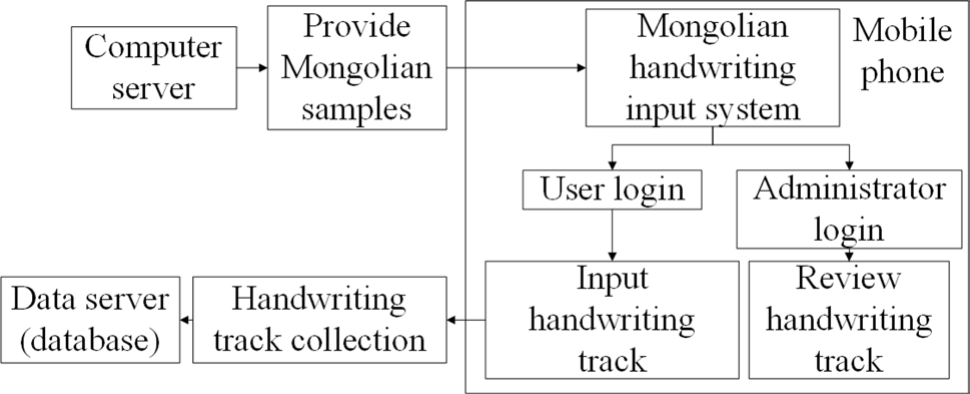
Architecture diagram of the handwriting trajectory acquisition system.

Owing to the popularity of smart phones, we decided to use a mobile phone touch screen to collect handwritten trajectories. Thus, a dedicated data collection application was developed. This application mainly enables the following tasks: data preparation, the real-time updating of data statistics, and data collection. The application is mainly divided into two parts: a background system and a user front end. The background system is mainly responsible for providing a Mongolian text template, collecting the user’s handwriting trajectory, and tracking the time of writing and the manual inspection results of each sample of text. The user front end is responsible for providing users with a handwriting environment and an environment for the manual inspection of text samples in the background. The overall architecture of the system is shown in Fig. 3. The role of the mobile phone is to act as the front-end environment for the system, divided into two main parts, one providing the writing environment for the users and the other providing the checking environment for the reviewers. Two different servers act as the back-end of the system, the computer sever provides the volunteers with samples to write, and the data sever provides the storage and reading of the writing traces.

**Figure 4:**
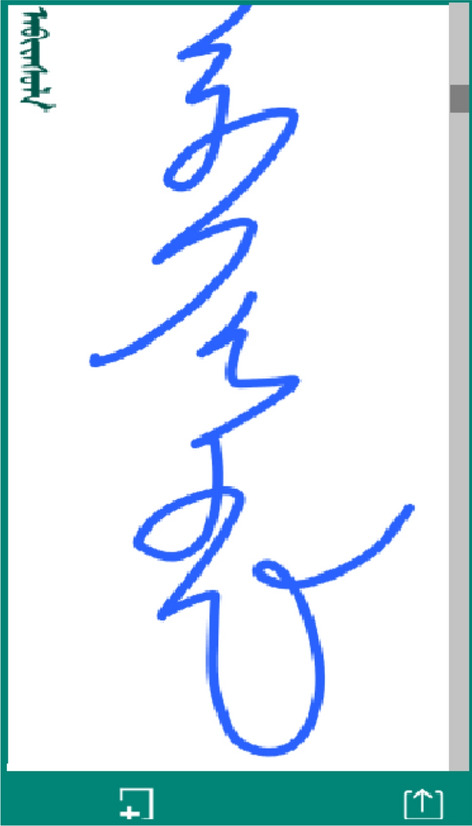
Main page of app.

When the user opens the Mongolian handwriting input application, they click the login button to access the handwriting interface shown in Fig. 4. The background assigns a target sample Mongolian word to the user. The text at the top left is the template word provided to the user. The user writes the corresponding word in the white area, and if the screen is not large enough to write the word, the user can use the scroll bar on the right to scroll down the drawing area. If the user is dissatisfied with their current writing sample, the user can use the clear button on the left to clear the writing area and rewrite the word. After writing, the user can click the submit button on the right to submit the written handwriting track to the data server. After submission, the application provides a new Mongolian word for the user to write.

### Data verification

**Figure 5:**
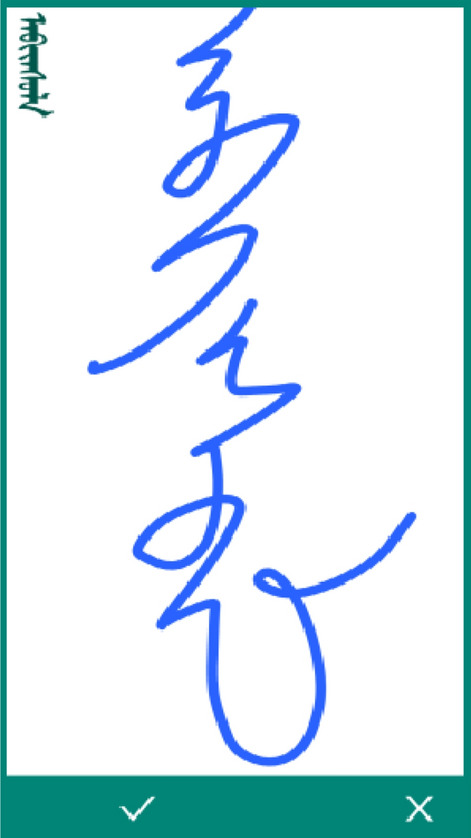
Review page of app.

To ensure the writing quality of Mongolian words, we set up several special expert reviewers who are native speakers of Mongolian. The goal of this operation was to delete misspelled or nonstandard writings. When an expert reviewer logs in to the application, the background sends a Mongolian text track written by the user to them for their manual inspection and approval. The application redraws the correct template word and the track written by the user on the screen. The reviewers choose to accept or reject the trajectory based on human eye comparison. Figure 5 shows the system operation diagram for the reviewers. The image in the top left corner of the Fig. 5 shows the standard writing method corresponding to the trajectory. When the reviewer determines that a sample meets the standard, select the check box option in the bottom left corner of the screen and the system will include this sample in the data set, otherwise select the fork option and the system will automatically jump to the review screen for the next sample. This system significantly reduces the cost of the inspection process.

We initially obtained about 210,000 handwritten trajectory samples, and after expert review, more than 160,000 samples remained. A large number of samples were deleted because, firstly, the volunteers had not been trained in writing standards and, secondly, they were uncomfortable with writing in Mongolian on mobile phones, resulting in sloppy or incomplete writing, thus causing some of the samples to be of poor quality.

### Dataset statistics

**Figure 6:**
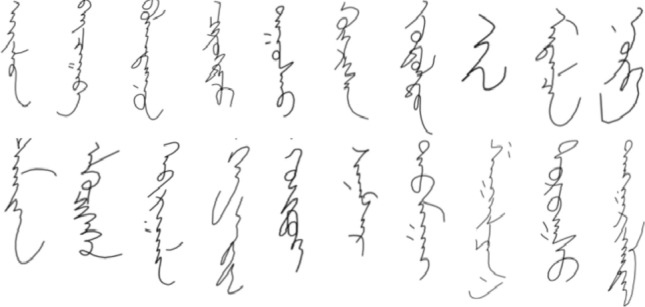
Some handwriting samples in MOLHW.

The MOLHW dataset is now publicly available at https://www.kaggle.com/fandaoerji/molhw-ooo to all researchers, and some examples of handwriting samples are shown in Fig. 6.

**Figure 7:**
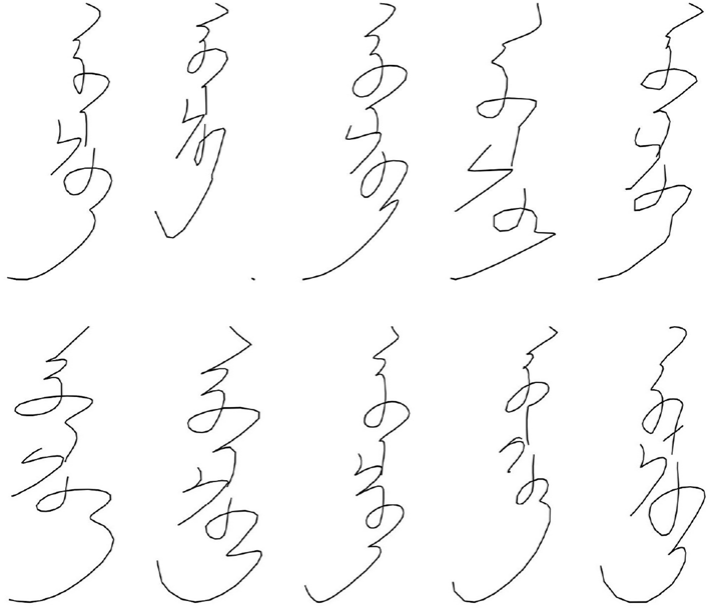
Some different handwriting style samples in MOLHW.

We show examples of different styles of writing in Fig. 7.

**Figure 8:**
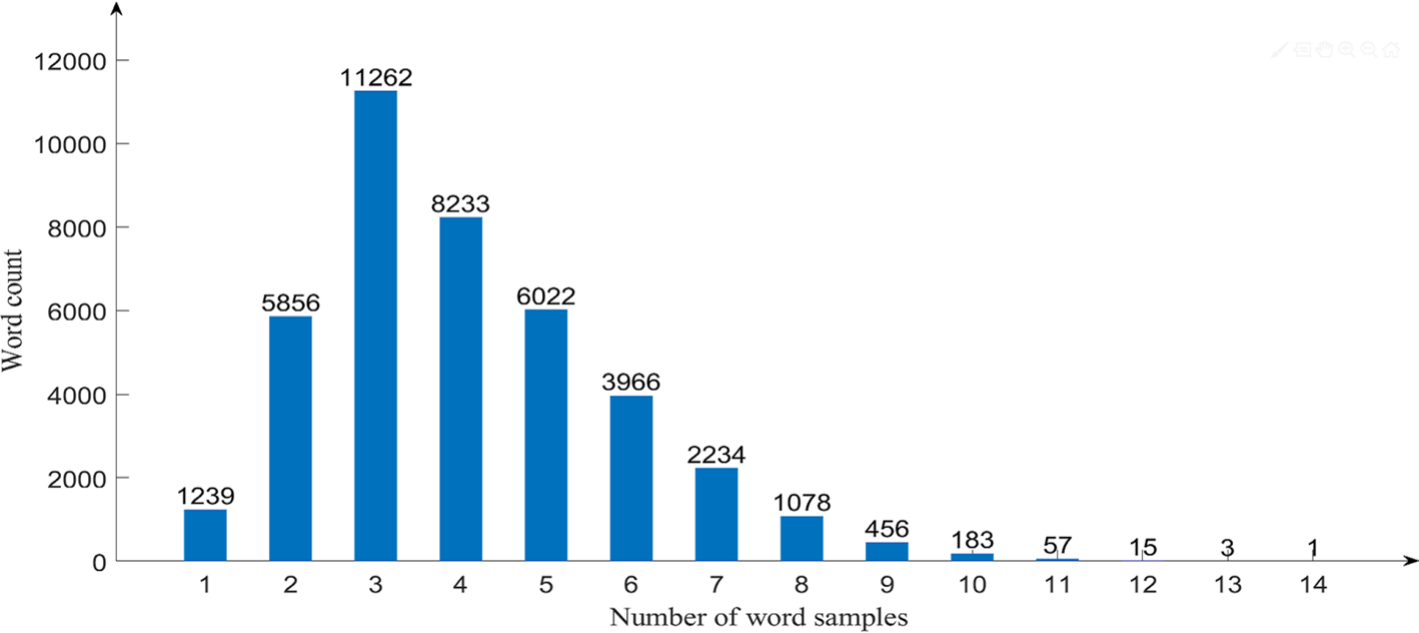
Word sample statistics.

The MOLHW dataset contains a total of 16,4631 handwriting samples and no separation of training and test sets. In this study, we randomly selected 70% as the training set, 20% as the test set, and the remaining 10% as the validation set. Users could divide the test set and training set according to their own needs. As multiple people were working in parallel, the words in the vocabulary were not written equally. The statistics of the handwriting sample collection of words are shown in Fig. 8.

It can be seen that 11,262 words were written three times, which was the most common frequency. The MOLHW dataset contains 40,605 words in total, which ensures that each word has at least one handwriting sample, the maximum number of collections is 14, and an average of four handwriting samples are collected per word. All 200 writing volunteers were freshmen in college, with ages ranging from 18 to 20, and the proportion of males and females was almost equal. The volunteer who wrote the most wrote 3374 samples, the one who wrote the second most wrote 2265 samples, the one who wrote the third most wrote 2246 samples, and the average number of writing samples per volunteer was 823.

The MOLHW dataset includes seven files: “MOLHW.txt”, “MOLHW_preprocess.txt”, “MOLHW_grapheme.txt”, “MOLHW_preprocess_grapheme.txt”, “ASCII2Unicode.txt”, “dict.txt”, and “grapheme_code.png”. The “MOLHW.txt” file is an original dataset file in text format, and each line is a handwriting sample in the format *[Label, Author ID, Screen width, Screen height, Screen pixel density, Writing track coordinates]*.

1. Label: Mongolian word in Latin transcription (case sensitive). The correspondence between Latin and Mongolian Unicode is explained in ASCII2Unicode.txt. For example, the label “abaci” can be converted to to “0x1820 0x182a 0x1834 0x1822” according to ASCII2Unicode.txt. The Mongolian word is.
2. Author ID: Writer md5 encrypted ID.
3. Screen width: Mobile phone screen width.
4. Screen height: Mobile phone screen height.
5. Screen pixel density: Mobile phone screen pixel density.
6. Writing track coordinates: The coordinate data arc is organized as [[x,y],[x,y],....,[x,y]]. The format of one pair of coordinates is “[x, y]”, where “x” represents the coordinate of the X-axis, and “y” represents the coordinate of the Y-axis. The upper left corner of the screen is marked as the origin of the coordinate system, moving the x-axis to the right and moving the y-axis down. The [$-1,-1$] in coordinates represents lifting a pen.

The “MOLHW_preprocess.txt” file is the dataset where the trajectory has been preprocessed, and the others are the same as “MOLHW.txt”. The file “MOLHW_grapheme.txt” is the dataset labeled grapheme codes, and the others are same as “MOLHW.txt” too. The file “MOLHW_preprocess_grapheme.txt” is the preprocessed dataset labeled grapheme codes, and the others are same as “MOLHW_preprocess.txt”. The file “dict.txt” is a Latin transliteration of Mongolian to grapheme code in the Mongolian dictionary file. The file “grapheme_code.png” is the grapheme code definition file.

## Benchmark evaluation

Mongolian online handwriting recognition can be considered a mapping process from a text trajectory sequence to a character sequence. In the Mongolian recognition task of this paper, based on the online Mongolian handwriting track data, the neural network recognizes the corresponding text, which can be considered as a sequence to sequence learning process. In the end-to-end task of variable-length sequences, the most widely used framework is encoder-decode structure, which is why in this paper we choose the encoder-decode framework structure to build the learning network as our baseline model. Another way of dealing with the variable length sequence problem is to use the CTC model, so in this paper we also combine the CTC network with the long short-term memory (LSTM) network in a comparative experiment. In total, three types of models were trained and validated. These are two encoder–decoder architectures. In the baseline model, we constructed an encoder–decoder^33^ based on an online Mongolian handwritten recognition model with an attention mechanism^34^. The second is a Transformer-based model, and the third is a LSTM with a CTC-based model. A full description of these three architectures is given in "Details of models" section. Finally, all the models were evaluated with a WER and character error rate (CER), where the CER is expressed as the average edit distance of words. Edit distance, also known as Levenshtein distance, is a quantitative measurement of the difference between two strings. It is measured by calculating how many times it takes to change one string into another.

### Preprocessing and framing

**Figure 9:**
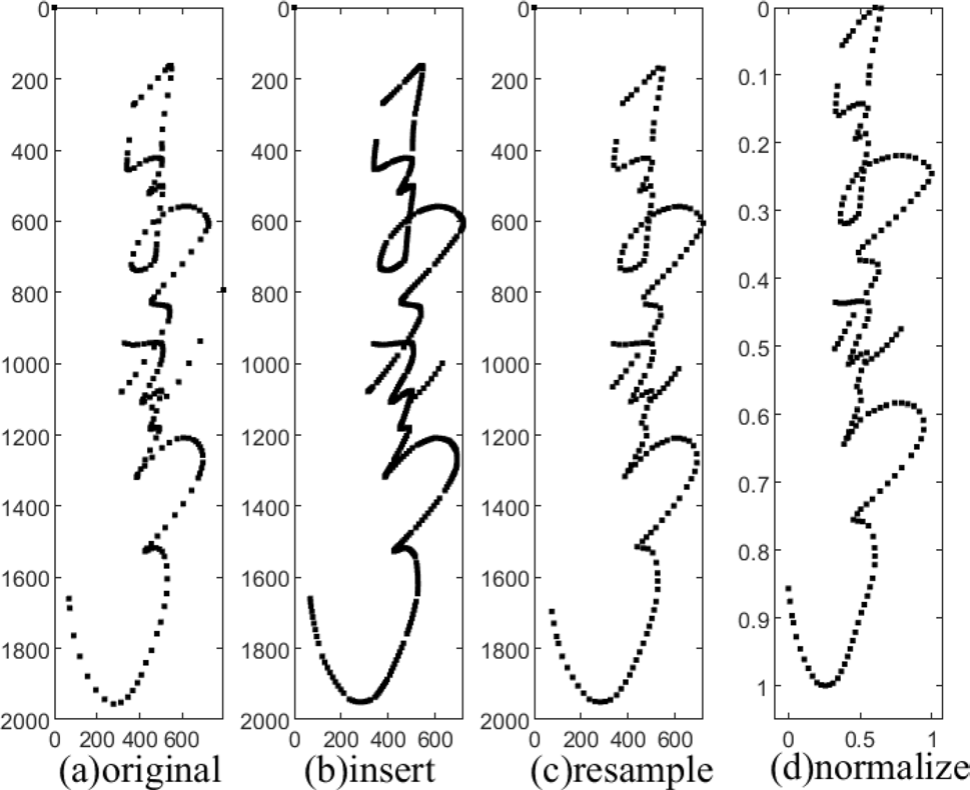
Trajectory data preprocessing.

To obtain a better recognition effect, the original writing track information needs some preprocessing operations. Owing to variations in writing speed, the acquired points were not distributed evenly along the stroke trajectory. Interpolation and resampling operations were used to recover missing data or force points to lie at uniform distances. Note that the image in the Fig. 9a was drawn from an original trajectory data. The detailed preprocessing steps are as follows:

1. Interpolation. Piecewise Bezier interpolation was used in the present study because it helps to interpolate points among a fixed number of points. This further helps distance the points at an equal interval. Figure 9a,b shows a comparison between the original trajectory data and the result of Bezier interpolation.
2. Resampling. After inserting the points, we resampled the samples according to the Euclidean distance between two adjacent points defined as the sampling distance. Figure 9b,c shows a comparison between the Bezier interpolation data and the trajectory data after resampling. The sampling distance is a parameter that needs to be adjusted.
3. Deletion. By screening the dataset for samples with less than a certain number of sampling points, a final threshold of 50 points was selected as the criterion for distinguishing whether a sample passed or failed, as we found that 50 points could be used as a threshold to screen out as much unqualified text as possible while removing as few correct samples as possible. We deleted the samples whose sampling point length was less than 50 after sampling.
4. Normalization. After the central axis of the sample data was translated to the X-axis, we normalized the sample on the XY-axis to ensure that the sampled handwritten text was in the center of the canvas. The final trajectory data are shown in Fig. 9d.

**Figure 10:**
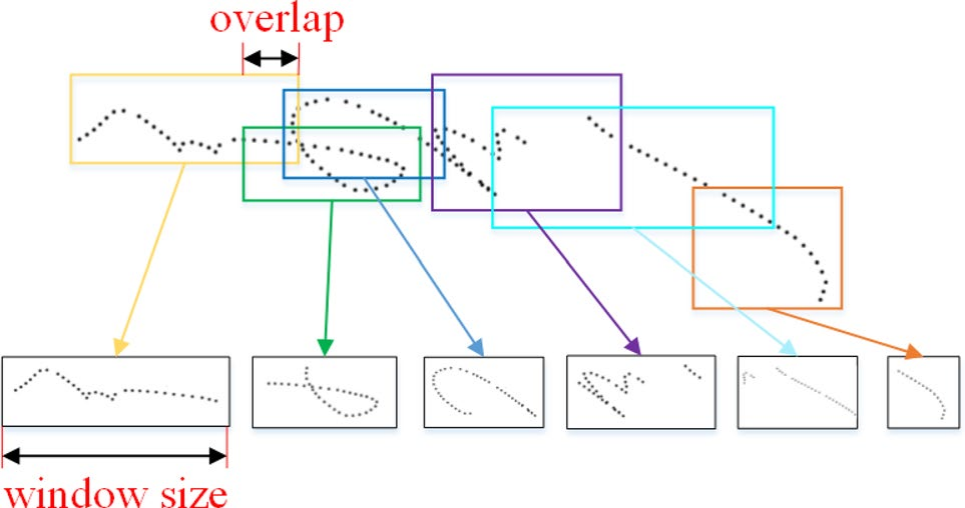
Trajectory image framing example.

The preprocessed text trajectory is a series of two-dimensional coordinates along the writing order. We used a sliding window to move along the writing order and concatenate all the coordinates within the window into a frame of data. A certain overlap was maintained when the window slid, and the framing process is shown in Fig. 10.

Both the sliding window size and the overlap length are in units of trajectory points, which are model hyperparameters that need to be tuned.

**Figure 11:**
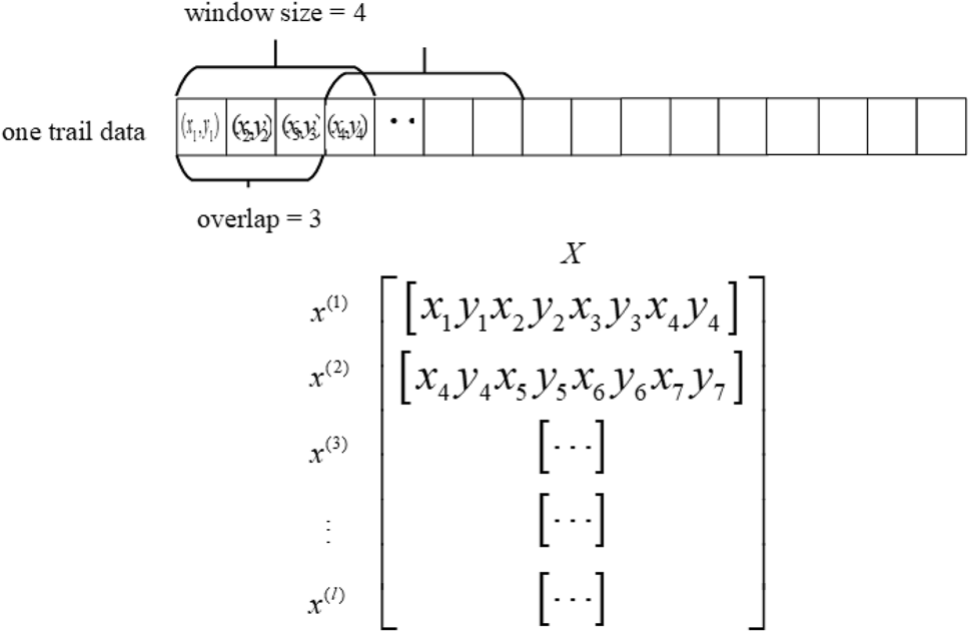
Trajectory data framing example.

The top row of data in Fig. 11 represents a sample trajectory, which identifies the range of a frame and the starting position of the next frame after a certain amount of offset. This figure is a diagram of framing for a window size of 4 and an overlap of 3. The matrix in Fig. 11 is the result of framing the sample, where each row represents a frame of data. This matrix is also the input data that is fed into the model.

### Details of models

#### Baseline model

**Figure 12:**
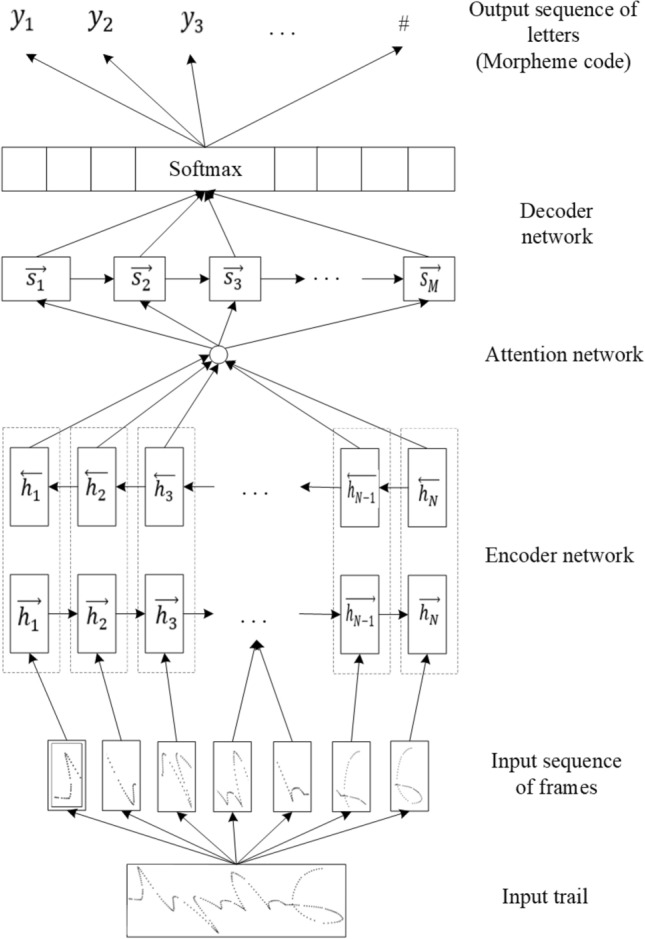
Architecture of the GRU based model.

**Figure 13:**
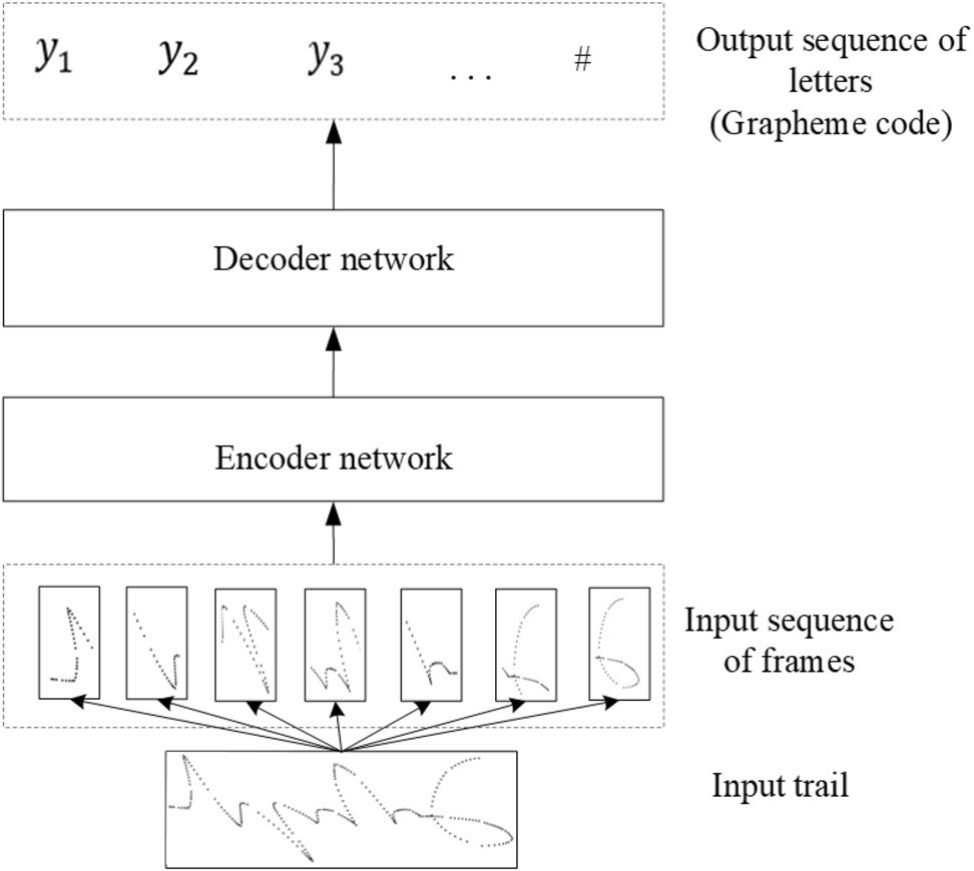
Architecture of the encoder–decoder model.

The proposed model was designed as a sequence-to-sequence architecture with an attention mechanism. This model is composed of a multi-layer BiGRU-based encoder and a GRU-based decoder, and a attention network is adopted to connect between the encoder and the decoder. Detailed diagram of the model for the specific encoder and decoder implementations of baseline model is shown in Fig. 12. The architecture is shown in Fig. 13.

The encoder is responsible for compressing the input sequence into a vector of a specified length, which can be regarded as the semantics of the sequence, while the decoder is responsible for generating the specified sequence according to the semantic vector. In this model, the sequence of frames from the handwritten trajectory is passed to the encoder and converted into hidden states of the corresponding encoder. Then, the last two hidden states of BiGRU are added up as the decoder’s initial hidden states. Next, at each step of the decoding process, the hidden states and encoder outputs are fed into the attention layer, in which an attention weight vector is calculated. Finally, the decoder receives the previous prediction output (initially, it feeds the start symbol SOS) and the attention context to generate a sequence of letters as the output of the model. The attention layer plays an important part in the proposed model because it allows the decoder to focus on different positions in a sequence of frames during decoding. In this model, the hidden layer size of GRU and the layer count of the encoder are hyperparameters that need to be tuned. The last layer of the decoder is a softmax classification layer, and the number of neurons depends on the number of target characters. When the Mongolian grapheme code is used as the target sequence, it contains a total of 52 neurons, and when Unicode is used, it contains a total of 36 neurons, including the end symbol EOS. In each step of decoding, the character with the highest probability is selected as the current output, and the decoding is stopped when EOS is encountered.

#### Transformer model

Similar to the proposed baseline model, the recognition model based on the transformer can also be regarded as being composed of an encoder and decoder and has been shown to be effective in numerous sequence-to-sequence problems. Thus, we built a Transformer model whose architecture is shown in Fig.13, and measured its performance on our dataset.

**Figure 14:**
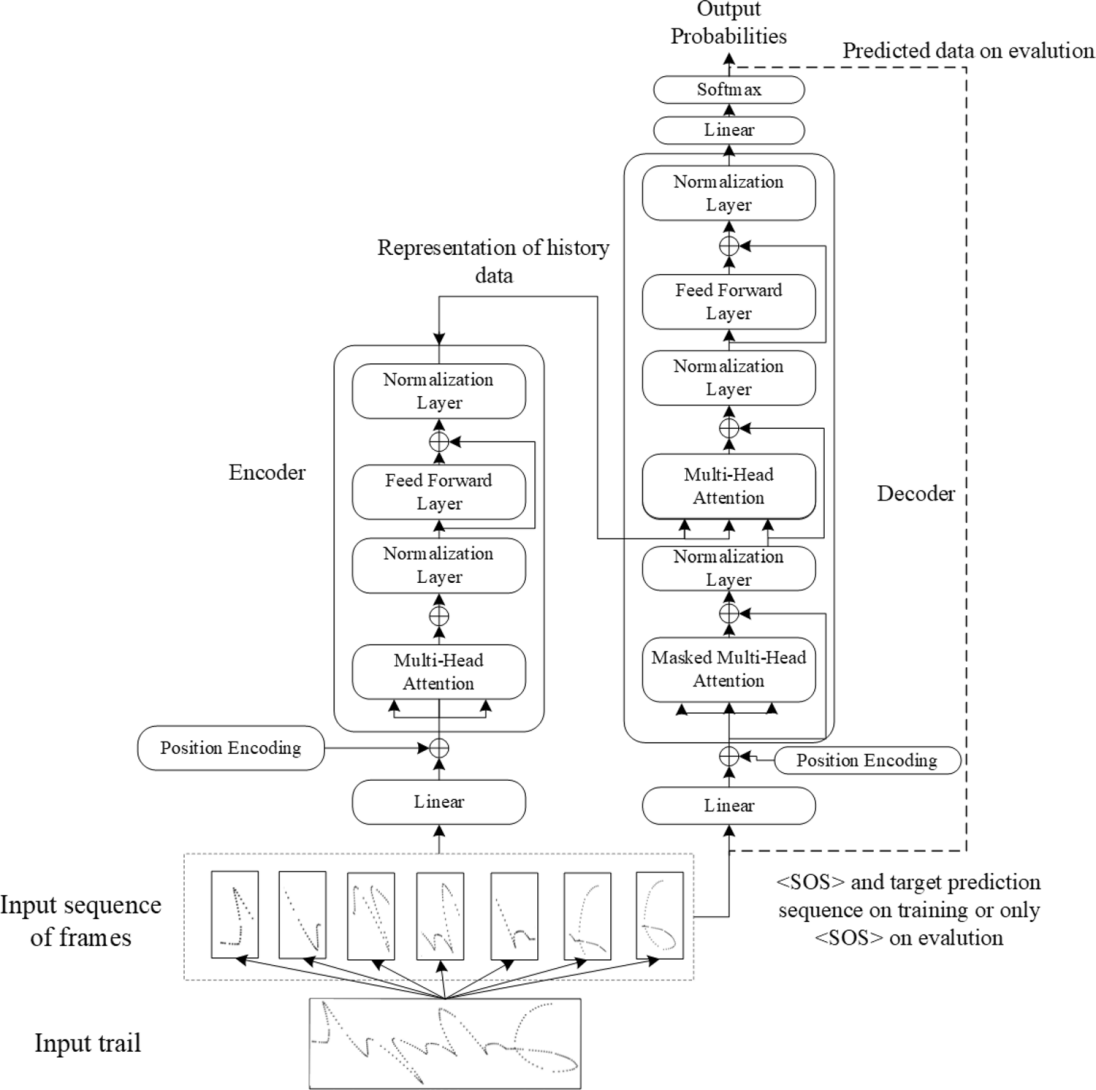
Architecture of the transformer model.

Vaswani et al.^35^ gave a specific description of the working principle of the transformer, which is not repeated here. Next, we focused on aspects related to understanding implementation of the transformer, and based on this, we built a simple model based on the transformer. Detailed diagram of the model for the specific encoder and decoder implementations of transformer model is shown in Fig. 14. Different from the baseline model, in the transformer, there are multiple sub-encoders with the same structure in the encoding module, and the input of each sub-encoder is the output of the previous sub-encoder. Each sub-encoder is composed of a self-attention mechanism and a feedforward neural network. The decoder has a structure similar to its encoder. In addition to the two sub-layers in each encoder layer, the decoder also inserts a third sublayer, which performs multiple attention on the output of the encoder stack. The experimental results of the baseline model confirm that the data characteristics contained in the raw data without any processing are insufficient. Therefore, the track data are divided into frames to make the characteristic information of the data more obvious. Then, the frame data are embedded, position-encoded, and sent to the encoder. After the transformer network, we predict one label at a time, while the cross-entropy loss function is applied in these situations.

#### LSTM-CTC model

**Figure 15:**
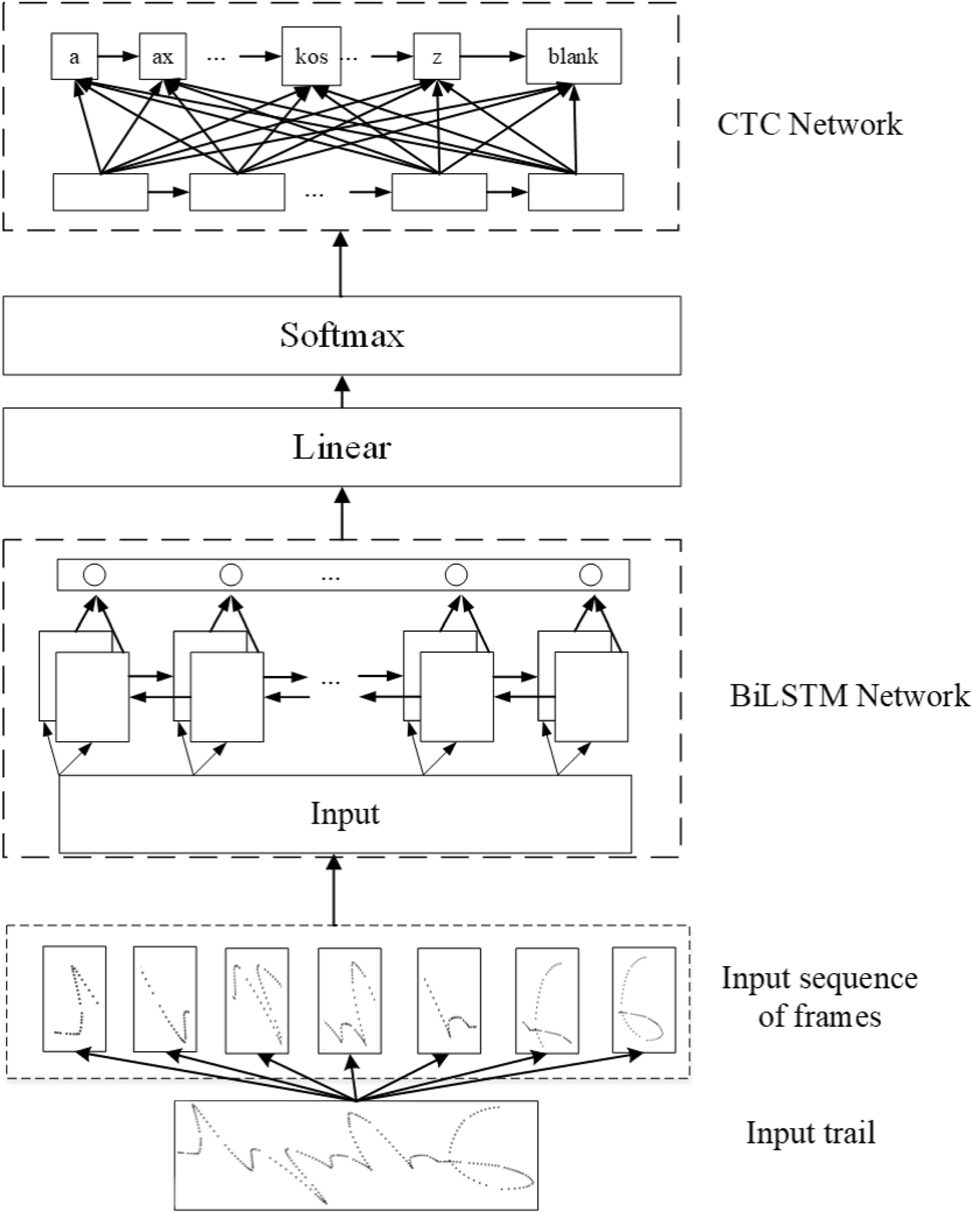
Architecture of the LSTM-CTC model.

LSTM is a type of recurrent neural network. LSTMs were developed to solve the vanishing and exploding gradient problems that plague many RNNs. Because LSTMs are much better at dealing with these two issues than earlier RNNs have been, LSTMs are suitable for tackling tasks that involve long-range dependencies in sequential data^32^. CTC is widely used in speech recognition, text recognition, and other fields to solve the problem where the lengths of input and output sequences are different and cannot be aligned. In our model, the CTC is actually our loss function. The LSTM with the CTC-based model consists of an LSTM network and a CTC network, as shown in Fig. 15. The LSTM network consists of a bidirectional LSTM (BiLSTM) layer with a 20% dropout rate to avoid overfitting. After the preprocessed frame data are sent to the BiLSTM layer, and this layer is followed by a fully connected layer and then a fully connected output layer of 52 classes, depending on the grapheme code classes, which has 51 letters and an empty character blank. After being normalized by the softmax layer, the output is sent to the CTC layer to calculate the loss with real labels of the grapheme code.

### Experimental results

Below, we give the experimental results of the optimization process of tunable parameters of the baseline model. The comparison results of the optimal performance of the three models are given at the end of this section. From the preprocessed MOLHW dataset, we randomly selected 70% as the training set, 20% as the test set, and the remaining 10% as the validation set. In all subsequent experiments, grapheme codes were used as target labels, but finally, we compared the performance of grapheme codes and Unicode under the same model. The cross-entropy loss function was used during the encoder–decoder training, and the training was stopped when the loss on the validation set was no longer reduced. Then, the epoch with the smallest loss on the validation set was selected as the optimal model. Each training epoch took nearly 270 second on the GPU NVIDIA Quadro P5000.

Before the model tuning, we carried out experiments with the same baseline model on the original data and the preprocessed data, with the fixed parameters selected as the sliding window size was 1, the overlap was 1, the hidden layer size was 64, and the number of encoder layers was 1. The corresponding experimental results are shown in Table 2 . The results in the table show that the preprocessed data performed better under the same model, so we carried out subsequent experiments on the preprocessed data.

**Table 2:** Original and preprocessed data experiment result.

Data	Train		Test	
	CER	WER (%)	CER	WER (%)
Preprocessed data	0.863	40.780	**0**.**903**	**43**.**635**
Original data	5.989	98.013	5.681	98.721

Significant values are in [bold].

As mentioned above, the sampling distance, sliding window size, overlap, hidden layer size, and number of encoder layers are all hyperparameters that had to be tuned. The tuning of hyperparameters adopted a simple search strategy. The first hyperparameter to be tuned was the sampling distance, and the search range was 3, 4, 5, and 6. When tuning, the other hyperparameters selected empirical values, and the sliding window size was 50, the overlap was 10, hidden layer size was 128, and the number of encoder layers was 1. The results are shown in Table 3. It can be seen from the table that the model had the best effect on the test set with a sampling distance of 4, so our subsequent experiments all sampled the dataset with a sampling distance of 4.

**Table 3:** Sampling distance tuning experiment result.

Sampling distance	Train		Test	
	CER	WER (%)	CER	WER (%)
3	0.810	39.096	0.996	46.546
4	0.730	35.013	**0**.**936**	**44**.**602**
5	0.698	31.204	0.965	44.959
6	0.909	42.905	1.113	51.326

Significant values are in [bold].

The second hyperparameter to be tuned was the sliding window size, and the search range was 15, 20, 30, 40, 50, and 60. When tuning, the other hyperparameters selected empirical values, the overlap was 20, the hidden layer size was 64, and number of encoder layers was 1. The experimental results are shown in Table 4. The experimental results show that the performance was best when the sliding window size was equal to 20. Next, we tuned the overlap hyperparameters, and compared the results when they were set to 4, 10, and 20. The results are shown in Table 5; the encoder–decoder network worked best when the overlap was 10.

**Table 4:** Sliding window size tuning experiment result.

Model parameter				Train		Test	
Window size	Overlap	Num layer	Hidden size	CER	WER (%)	CER	WER (%)
15	10	1	64	1.361	58.044	1.375	60.219
20				1.054	47.134	**1**.**129**	**51**.**096**
30				1.105	48.893	1.184	52.538
40				1.194	51.580	1.261	54.769
50				1.120	49.582	1.190	53.439
60				1.289	55.283	1.332	58.233

Significant values are in [bold].

**Table 5:** Overlap tuning experiment result.

Model parameter				Train		Test	
Overlap	Window size	Num layer	Hidden size	CER	WER (%)	CER	WER (%)
10	20	1	64	1.054	47.134	1.129	51.096
5				0.849	39.291	**0**.**925**	**43**.**200**
2				0.899	41.600	0.935	44.076

Significant values are in [bold].

The optimal values of the three hyperparameters of sampling distance, sliding window size, and overlap in the data preprocessing process were determined, and their values were 4, 20, and 10, respectively. In the following experiments, we searched hidden layers of sizes 64, 128, and 256, and searched encoder layers 1, 2, 3, and 4 simultaneously. The results are shown in Table 6. It can be seen that with the increase of the number of layers, the recognition effect gradually improved, but after reaching four layers, the increase was no longer obvious. When the number of layers was one and two, as the size of the hidden layer increased, the recognition performance also improved, but when the number of layers was three, the performance decreased when the size of the hidden layer reached 256. The best performance was achieved when the number of layers was three and the hidden layer size was 128, with a CER of 0.471 and a WER of 24.281% on the test set.

**Table 6:** Num layer and hidden size tuning experiment result.

Model parameter				Train		Test	
Window size	Overlap	Num layer	Hidden size	CER	WER (%)	CER	WER (%)
20	5	1	64	0.849	39.291	0.925	43.200
			128	0.641	31.566	0.811	38.756
			256	0.595	29.611	0.767	37.005
		2	64	0.434	23.296	0.591	29.934
			128	0.404	21.751	0.578	29.237
			256	0.366	19.729	0.569	28.508
		3	64	0.303	16.857	0.472	24.862
			128	0.232	13.515	**0**.**471**	**24**.**281**
			256	0.286	15.926	0.506	25.436
		4	64	0.341	18.790	0.486	25.406

Significant values are in [bold].

At the end of the experiment, we compared the performance of grapheme codes and Unicode. We used Unicode as the label and repeated the experiment, in which the model parameters selected the optimal parameters of the grapheme code, and the results are shown in Table 7. It can be seen that the performance of grapheme code was much higher than that of Unicode encoding, in which CER was reduced by 0.309, and WER is reduced by 17.437% on the test set.

**Table 7:** Experimental results of Unicode and grapheme code comparison.

Label	Train		Test	
	CER	WER (%)	CER	WER (%)
Grapheme code	0.232	13.515	**0**.**471**	**24**.**281**
Unicode	0.580	32.787	0.780	41.718

Significant values are in [bold].

With the preprocessed data, the first three rows of Table 8 shows the recognition accuracy based on three models. In terms of WER and CER, the transformer model performed much better than our baseline model, with a 7.312% increase in the WER rate and a 0.098% increase in the CER on the test set. Our results again confirm the excellent performance of the Transformer structure-in-sequence to sequence problem. We tested the performance of the original data with the three models, and the results are shown in the last three rows of Table 8 . In overall comparison, the preprocessed data showed strong learnability for all three models which shows that our pre-processing was very effective.

**Table 8:** Experimental results of different model comparison.

Data	Model	Train		Test	
		CER	WER (%)	CER	WER (%)
Preprocessed data	Baseline model	0.232	13.515	0.471	24.281
	LSTM-CTC	0.347	21.432	0.528	30.161
	Transformer	0.171	8.170	**0**.**373**	**16**.**969**
Original data	Baseline model	0.796	36.048	0.884	40.533
	LSTM-CTC	0.915	48.884	1.048	51.836
	Transformer	2.485	91.056	2.583	91.486

Significant values are in [bold].

### Error analysis

**Figure 16:**
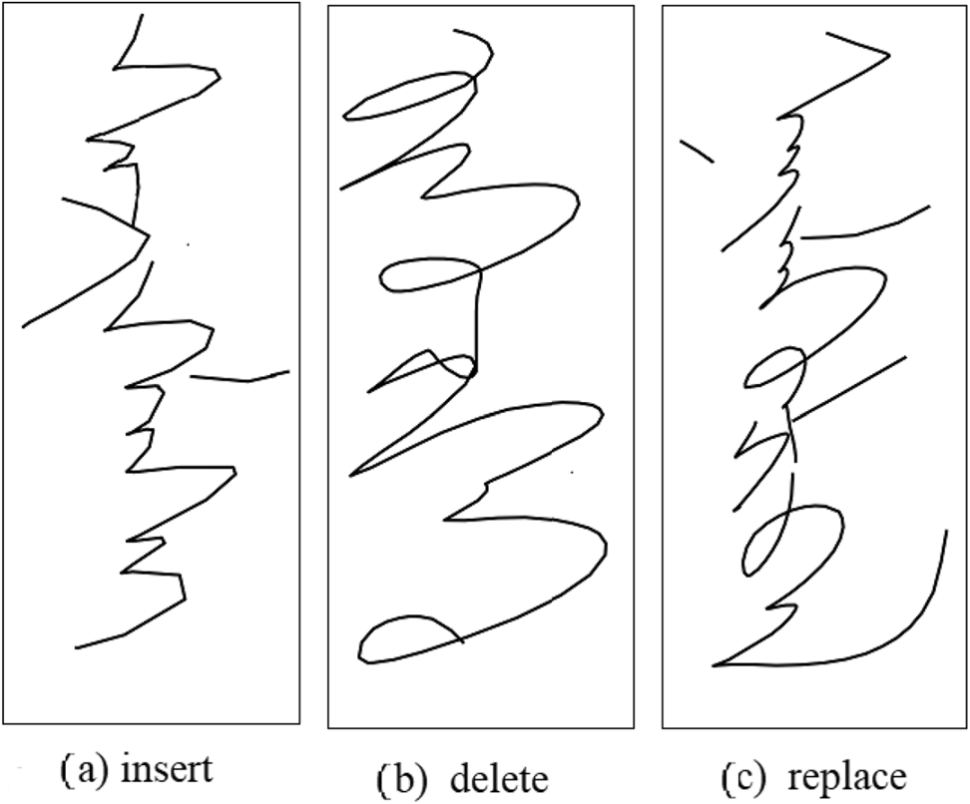
Incorrectly recognized images.

For the experimental results of the our baseline model, we give the error analysis on the test set. The number of samples in the test set was 32,926, and the WER was 24.81%; that is, 8168 samples had recognition errors. Figure 16 shows the images with recognition errors caused by different error types. Figure 16a shows that the corresponding true label is ‘s az jz iz gzz lz az s ix’, and owing to the insertion of ‘hes bax’, the wrong decoding result of the model is ‘s az jz iz gzz lz az s hes bax ix’. Figure 16b shows that the corresponding true label is ‘a o iz gzz hes box c iz hes bax hes box’, and because of the lack of ’gzz,’ the wrong decoding result of the model is ‘a o iz hes box c iz hes bax hes box’. Figure 16c shows that the corresponding true label is ‘n az iz iz lz az hes box lz c iz bos bax lx’, and because ‘hes’ replaces ‘bos’, the wrong decoding result of the model is ‘n az iz iz lz az hes box lz c iz hes bax lx’.

For the above three errors, the statistics of recognition errors are shown in Table 9. As we can see from the table, the most common occurrence in the recognition process is repalce errors, meaning that a correct character is replaced by another incorrect character. This is followed by deletion errors, where a character is not recognised, resulting in a character being missing from the recognition result, and finally insertion errors, where a single character is incorrectly recognised as more than one character, resulting in an extra character in the recognition result.

**Table 9:** Incorrectly recognized images.

Error classification	Times
Replace	4242
Delete	2367
Insert	1559

We analyzed the most common errors, replace error. The statistics of the test set replace error results are given in Table 10.

**Table 10:** Statistics of replace error.

Times	Original char	Shape	Wrong char	Shape
336	ix		ax	
288	lx		ax	
216	mx		lx	
191	ax		ix	
190	ax		lx	

As can be seen from the table, the character that was most likely to be replaced and replaced was ‘ax’. Because of its simple structure, it is easily confused with other similar characters in the case of uneven data coordinates.

## Conclusion

A dataset (MOLHW) consisting of handwritten Mongolian words was described in this paper. The dataset contained 164,631 samples of Mongolian online text with 40,605 Mongolian words written by 200 writers. To evaluate the MOLHW dataset, we outlined, implemented, and trained three models. An attention-based encoder–decoder model used for an online handwritten Mongolian character recognition model network was used as the baseline model to make a preliminary evaluation of our dataset. The experiment results show that our model could effectively recognize the corresponding grapheme code sequences from the continuous coordinate sequences of Mongolian words handwritten online, which can effectively solve the problems of primitive segmentation and OOV word recognition, which are caused by Mongolian being an agglutinative language. Subsequently, experiments were carried out on our dataset based on LSTM-CTC and the Transformer model. Our results confirm the excellent performance of the transformer structure in sequence to sequence problem, which obtained the best experimental results, with a 16.969% WER on the test set. Thus, we believe that the MOLHW dataset can be used as a benchmark dataset for studies related to Mongolian online handwriting recognition, writer identification, handwritten text generation, and related areas. This database is freely available upon request for interested researchers.

## Data Availability

All relevant codes included in this study are available upon request by contacting with the corresponding author. And the MOLHW dataset is now publicly available at https://www.kaggle.com/fandaoerji/molhw-oootoallresearchers.
